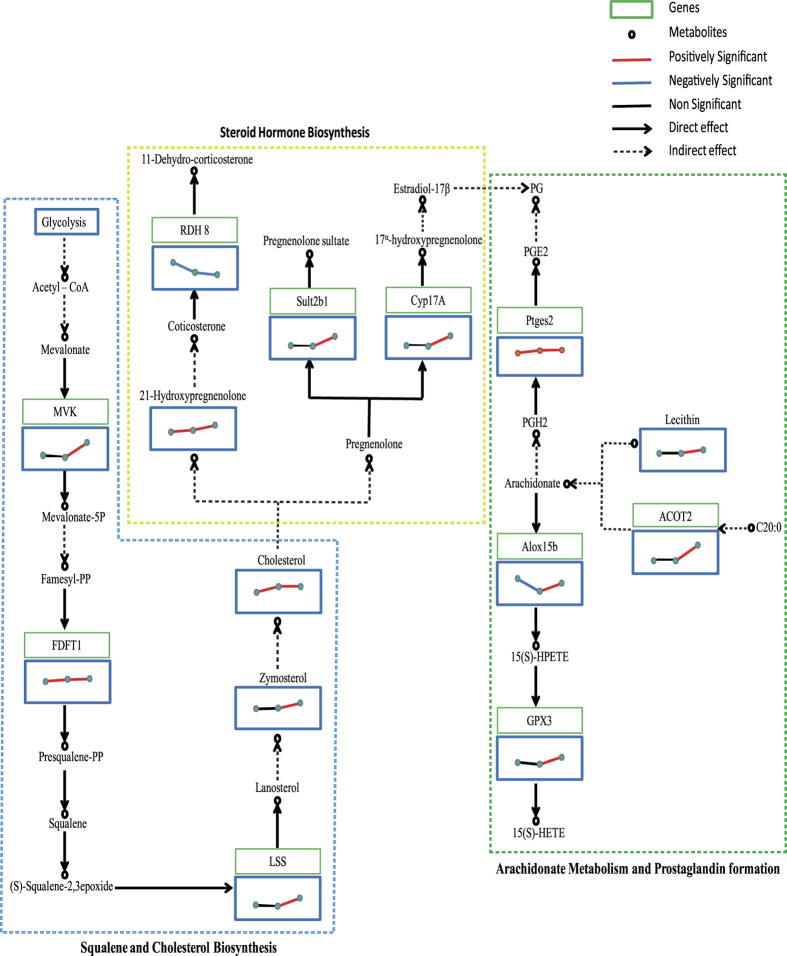# Integrated application of transcriptomics and metabolomics yields insights into population-asynchronous ovary development in *Coilia nasus*

**DOI:** 10.1038/srep31835

**Published:** 2016-08-22

**Authors:** Gangchun Xu, Fukuan Du, Yan Li, Zhijuan Nie, Pao Xu

**Affiliations:** 1 Wuxi Fisheries College, Nanjing Agricultural University, Wuxi, Jiangsu, 214081, China; 2 Key Laboratory of Freshwater Fisheries and Germplasm Resources Utilization, Ministry of Agriculture, Freshwater Fisheries Research Center, Chinese Academy of Fishery Sciences, Wuxi, Jiangsu, 214081, China

## Abstract

Populations of *Coilia nasus* demonstrate asynchronous ovarian development, which severely restricts artificial breeding and large-scale cultivation. In this study, we used a combination of transcriptomic and metabolomic methods to identify the key signaling pathways and genes regulation affecting ovarian development. We identified 565 compounds and generated 47,049 unigenes from ovary tissue. Fifteen metabolites and 830 genes were significantly up-regulated, while 27 metabolites and 642 genes were significantly down-regulated from stage III to stage IV of ovary development. Meanwhile, 31 metabolites and 1,932 genes were significantly up-regulated, and four metabolites and 764 genes were down-regulated from stage IV to stage V. These differentially expressed genes and metabolites were enriched by MetScape. Forty-three and 50 signaling pathways had important functions from stage III–IV and from stage IV–V in the ovary, respectively. Among the above signaling pathways, 39 played important roles from ovarian stage III–V, including “squalene and cholesterol biosynthesis”, “steroid hormone biosynthesis”, and “arachidonate metabolism and prostaglandin formation” pathways which may thus have key roles in regulating asynchronous development. These results shed new light on our understanding of the mechanisms responsible for population-asynchronous development in fish.

Ovarian development in most teleosts can be classified as synchronous or asynchronous, according to the growth pattern of the oocytes in the ovary at any one time^1^,^2^. In synchronous spawners, such as in the brown trout (*Salmo trutta*)^3^, egg production can be an annual event, or a single occurrence in the individual’s lifetime, as in the Pacific pink salmon (*Oncorhynchus gorbuscha*)^4^. In asynchronous spawners, eggs are recruited from a heterogeneous population of developing oocytes and are subsequently ovulated in several batches during each spawning season^1^. A number of teleosts are asynchronous batch spawners, including the sea bass (*Centropristis striata*)^5^, cod (*Gadus morhua*)^6^, and halibut (*Hippoglossus hippoglossus*)^7^. Some species, such as haddock (*Melanogrammus aeglefinus*), are unusual in ovulating a number of times during the breeding season, but rather than having a heterogeneous array of different-sized oocytes, they exhibit more or less synchronous oocyte growth up to the preoocyte maturation stage, and then produce several batches of eggs at intervals of 2–5 days^1^. Ovary development in the estuarine tapertail anchovy (*Coilia nasus*, junior synonym *C. ectenes*) is similar to that in haddock.

*C. nasus* is widely distributed in the Yangtze River, the coastal waters of China and Korea, and the Ariake Sound in Japan^8^. *C. nasus* is a migratory fish, juveniles feed and develop in the sea^8^, during which time the ovary develops to stage II by the middle of October and remains at this stage until February the following year, when the fish migrate to the Yangtze River. The ovary continues to develop during migration, and spawning occurs gradually during this process. Although ovary development in *C. nasus* is synchronous and each individual produces several batches of eggs at intervals of 1–2 days, the population-spawning period is prolonged, from April to October. This is because the population structure of *C. nasus* is complex, including fish with different stages of ovary development in the same place at the same time. Spawning thus occurs over a long period in *C. nasus*. After spawning, the ovary is gradually restored to stage II, and the fish then migrate down the river and back into the sea in batches. This system is referred to as population-asynchronous ovary development, in which development is synchronous within individuals, but asynchronous among different individuals within the same population. However, this feature severely restricts their large-scale cultivation and artificial breeding. In this study, we used combined transcriptomic and metabolomic methods to identify the key signaling pathways and gene expression profiles involved in regulating ovarian development.

Rapid progress in next-generation sequencing technologies has made them suitable for large-scale efficient and economical production of expressed sequence tags. Transcriptome sequencing facilitates functional genomic studies, including global gene expression, novel gene discovery, and the assembly of full-length genes^9^,^10^,^11^,^12^. However, the obtained results have been too global, making it difficult to determine the key pathways responsible for regulating specific traits. We therefore performed an integrated study involving transcript profiling together with the analysis of primary metabolites to improve our understanding of the regulation of ovarian development. We examined changes in metabolite levels and determined if they were consistent with changes in transcript abundance during ovary development from stage III to stage V.

## Materials and Methods

### Experimental animals

Three 60.0 m × 20.0 m × 2.0 m ponds were each stocked with 1000 juvenile *C. nasus*. The fish were acclimated to the ponds for approximately 3 years before the experiments. The stocking density was 37.5 g/m^2^, and 71.0% of the fish survived for the experiment. Seventy fish in each pond were euthanized to examine and analyze ovarian development. Excess fish were stocked in ponds in order to ensure the availability of adequate numbers of fish. *C. nasus* spawning was studied in 2014, and gonad samples were collected at the peak of spawning. The fish were euthanized with 70 mg/L buffered tricaine methanesulfonate (MS-222), and immediately submerged in crushed ice to retard degradation of RNA. All fish appeared healthy during dissection. Ovaries were removed, placed in liquid nitrogen, and stored at −80 °C for subsequent analysis. The mean length (±standard error) was 286.76 ± 12.24 mm and the mean mass was 101.83 ± 12.82 g for all fish (n = 26) sampled in this experiment.

### RNA sequencing, assembly, and annotation

Transcriptome sequencing was carried out on an Illumina HiSeq 2000 platform that generated about 100 bp paired-end raw reads (BGI, Shenzhen, China). After removing adaptor sequences, ambiguous ‘N’ nucleotides (with a ratio of ‘N’ >5%), and low-quality sequences (quality score <10), the remaining clean reads were assembled using trinity software^13^, as described for *de novo* transcriptome assembly without a reference genome. Homology annotation was performed as described by Du *et al*.^14^.

### Analysis of differentially expressed genes

To analyze stress-responsive, differentially expressed genes in *C. nasus*, the number of reads for each of the contigs from the two samples was converted to reads per kilobase per million (RPKM)^15^. The MA-plot-based Method with Random Sampling model (MARS) in the DEG seq package was then used to calculate the expression abundance of each contig in the analyzed samples^16^. We determined the P-value threshold using a false discovery rate (FDR), with an FDR value <0.001 considered to indicate a significant expression abundance.

Pathway-enrichment analysis identifies significantly enriched metabolic pathways or signal transduction pathways represented by differentially expressed genes, compared with the whole genome background. Calculations were made using Bonferroni adjustments^17^. After multiple testing corrections, we considered pathways with a Q value ≤0.05 as significantly enriched among the differentially expressed genes. Q value is defined as the FDR analog of the P value. The Q value of an individual hypothesis test is the minimum FDR at which the test may be called significant.

### Metabolomics detection

A total of 0.05 g of each sample in a 2 ml eppendorf tube was extracted with 0.4 ml methanol–chloroform (v/v, 3/1), followed by the addition of 20 μl of L-2-chlorophenylalanine (1 mg/ml stock in dH_2_O) as an internal standard, homogenization in a ball mill for 3 min at 65 Hz, and centrifugation at 12,000 rpm for 15 min at 4 °C. The supernatant (approximately 0.38 ml) was then transferred to a 2 ml glass vial. A total of 15 μl of each sample was mixed in a 2 ml gas chromatography–mass spectrometry (GC–MS) vial and analyzed for quality control.

The extracts were dried in a vacuum concentrator for about 2 h at 37 °C, followed by the addition of 80 μl methoxylamine hydrochloride (dissolved in pyridine, final concentration 20 mg/ml) to the dried metabolites, and incubation at 80 °C for 20 min in an oven, after mixing and sealing. The lid was opened and 100 μl bis-trimethylsilyl trifluoroacetamide, containing 1% trimethylchlorosilane (v/v) was added to each sample, which was sealed again and incubated at 70 °C for 1 h. A volume of 5 μl FAMEs (standard mixture of fatty acid methyl esters, C8–C16: 1 mg/ml; C18–C24: 0.5 mg/ml in chloroform) was added to the mixed sample, cooled to room temperature, and mixed well for GC–time-of-flight (TOF)–MS analysis.

GC–TOF–MS analysis was performed using an Agilent 7890 gas chromatograph system coupled with a Pegasus HT time-of-flight mass spectrometer. The system utilized a Rxi-5Sil MS column (30 m × 250 μm inner diameter, 0.25 μm film thickness; Restek, Bellefonte, PA, USA). A 1 μl aliquot of the analyte was injected in splitless mode. Helium was used as the carrier gas, the front inlet purge flow was 3 ml/min, and the gas-flow rate through the column was 20 ml/min. The initial temperature was kept at 50 °C for 1 min, then raised to 330 °C at a rate of 10 °C per min, then kept at 330 °C for 5 min. The injection, transfer line, and ion source temperatures were 280, 280, and 250 °C, respectively. The energy was −70 eV in electron impact mode. The mass spectrometry data were acquired in full-scan mode with a m/z range of 30–600 at a rate of 20 spectra per s after a solvent delay of 366 s.

Chroma TOF4.3X software from LECO Corporation and the LECO-Fiehn Rtx5 database were used for raw peak exacting, data baseline filtering and calibration, peak alignment, deconvolution analysis, peak identification, and integration of the peak area^18^. The retention time index method was used for peak identification, with a tolerance value of 5000.

### Analysis of differential metabolomics

The resulting three-dimensional data involving the peak number, sample name, and normalized peak area were fed into the SIMCA14 software package (Umetrics, Umea, Sweden) for principal component analysis (PCA) and orthogonal projections to latent structures–discriminate analysis (OPLS–DA). PCA showed the distribution of the original data. OPLS-DA was applied to obtain a higher level of group separation and a better understanding of the variables responsible for classification. The classification parameters from the software were R^2^Y = 0.961 and Q^2^Y = 0.467, which were stable with adequate goodness-of-fit and predictive ability. Seven-fold cross validation was used to estimate the robustness and predictive ability to further validate the model. The R^2^ and Q^2^ intercept values were 0.906 and −0.0636 after 200 permutations. The low values of the Q^2^ intercept indicated the robustness of the models and indicated a low risk of over-fitting and good reliability. A loading plot was constructed based on the OPLS-DA, which showed the contribution of the variables to the differences between two groups and the important variables situated far from the origin; however, the loading plot was complex because of the many variables. We refined this analysis by obtaining the first principal component of variable importance projection values, with values >1.0 first selected as changed metabolites^19^. In step 2, the remaining variables were then assessed using Student’s *t*-test, and variables with P > 0.05 between the two groups were discarded^19^.

### Animal welfare

All procedures were carried out in accordance with the Guide for the Care and Use of Laboratory Animals (Ministry of Science and Technology of China, 2006), and all experimental protocols were approved by the animal ethics committee of the Chinese Academy of Fishery Sciences.

## Results

### Spawning behavior in *C. nasus*

**Figure 1:**
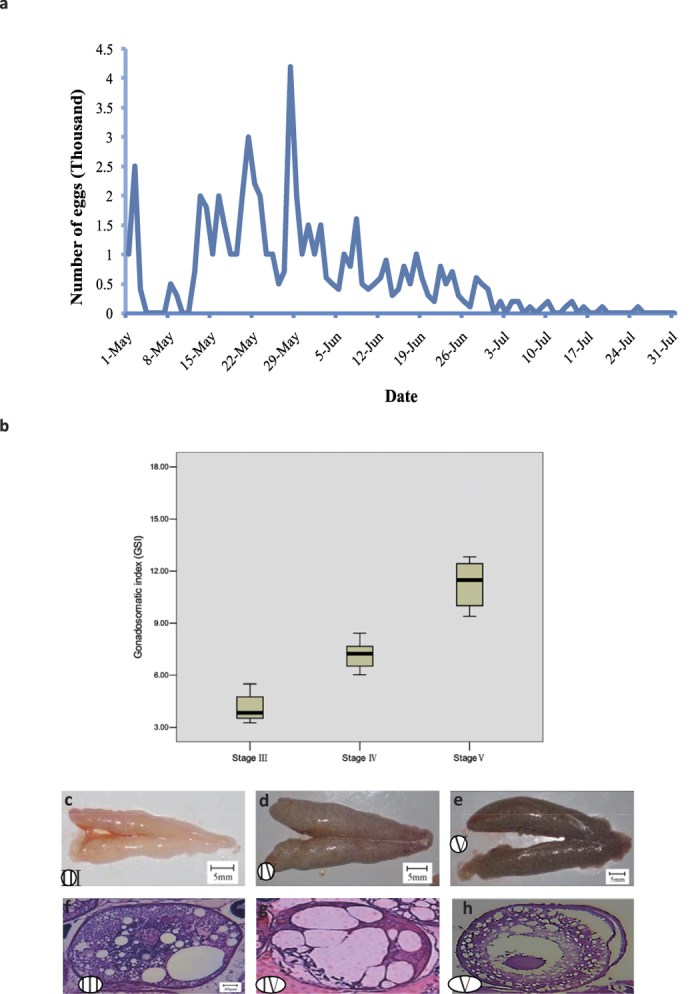
*C. nasus* ovarian development. (**a**) Spawning times of breeding *C. nasus*. (**b**) Gonadosomatic indexes of *C. nasus*. (**c**–**e**) Anatomical features of ovarian development in *C. nasus*: (**c**) stage III; (**d**) stage IV; (**e**) stage V. (**f**–**h**) Germ cells at different stages of ovarian development in *C. nasus:* (**f**) stage III; (**g**) stage IV; (**h**) stage V. Paraffin-embedded tissue sections stained with hematoxylin and eosin. All images ×40.

We studied the spawning behavior of *C. nasus* in 2014. Peak spawning occurred between May and June (Fig. 1a), with spawning concentrated around 20:00–20:30, and the fertilization rate was 80–92%. A total of 569,000 eggs were collected during the study. Water temperature is a key factor controlling *C. nasus* spawning, with an optimal temperature of 20–28 °C. In May and June, the highest spawning peak occurs when the temperature remains at 25–28 °C for a period of 5 days. Based on these observations, we collected samples around the spawning peak (Fig. 1b). We prepared paraffin tissue sections with hematoxylin and eosin staining to investigate the developmental condition of the ovary (Fig. 1c–h). Based on the above results, we selected eight stage III, ten stage IV, and eight stage V samples for transcriptomic and metabolomic analysis.

### GC–MS analysis of metabolites in different-stage ovaries in *C. nasus*

Representative chromatograms of homogenized ovary samples analyzed by GC–MS are shown in Fig. S1. A total of 565 compounds were identified in the ovary homogenates (Table D1), mostly fatty and amino acids involved in lipid peroxidation, energy metabolism, and amino acid metabolism. All data on retention time, exact mass, and peak intensity were recorded for multiple statistical analyses, including PCA and OPLS-DA. These analytical methods were chosen because of their ability to cope with highly multivariate, noisy, collinear, and possibly incomplete data.

The GC–MS results were displayed as “score plots” by PCA, which represent the distribution of the samples in multivariate space. PCA score plots were obtained from the GC–MS data for the three ovarian-development stages, and showed that the three groups exhibited tendencies to be different from each other, indicating their different metabolic profiles (Fig. S2a). OPLS-DA was conducted to determine if the different ovarian development stages influenced the metabolic pattern. The score plots (Fig. S2b,c) for the OPLS-DA model showed clear separation among the different groups. All samples fell within the 95% confidence interval. These results confirmed that the different development stages were represented by different metabolic patterns.

### Generation and *de novo* assembly of *C. nasus* transcriptome date

We performed transcriptome sequencing of three libraries from gonad samples of *C. nasus* using an Illumina HiSeq 2000 platform sequencer, which generated 65.75, 65.75, and 65.70 million reads, respectively, from the three libraries. After removing low-quality and short reads, 197,200,000 clean reads corresponding to mRNAs were obtained, covering 19,710,000,000 bases (Table S1).

We generated a total of 47,049 unigenes using the Trinity assembly program (Table S2). The mean unigene size and N50 were 1,358 bp and 2,472 bp, respectively. More than half of the unigenes (31,767; 67.5%) were ≥500 bp, and 5,647 unigenes were >3,000 bp in length (Fig. S3).

### Functional annotation and pathway assignment

A total of 47,049 unigenes were annotated according to seven databases: Non-redundant protein (Nr), Non-redundant nucleotide (Nt), Swissprot, Kyoto Encyclopedia of Genes and Genomes (KEGG), Cluster of Orthologous Groups of proteins (COG), Interpro, and Gene Ontology (GO) (Fig. S4 and Table S3). GO, which is an international standardized gene-function classification system, classified 6,735 non-redundant unigenes into three major functional categories (“biological process”, “cellular component”, and “molecular function”) and 59 subcategories (Fig. S5). Of the sequences categorized as “biological process”, the dominant subcategories were “cellular process” (3,771, 60.0%) and “metabolic process” (3,222, 47.8%). In the “cellular component” category, “cell” (2,982, 44.3%) and “cell part” (2,982, 44.3%) were the most highly represented, followed by “organelle” (2,021, 30.0%) and “membrane” (1,559, 23.1%). Among the “molecular function” terms, there was a significant proportion of clusters assigned to “binding” (3,496, 51.9%) and “catalytic activity” (2,745, 40.8%). However, within each of the three categories, few genes were assigned to the subcategories “cell aggregulation”, “nucleoid”, and “protein tag”.

To classify orthologous gene products, 13,904 (29.6%) non-redundant unigenes were subdivided into 25 COG classifications. Among them, the cluster of “general function prediction only” (4,711, 33.9%) represented the largest group, followed by “cell cycle control, cell division, chromosome partitioning” (2,826, 20.3%), “translation, ribosomal structure and biogenesis” (2,689, 19.3%), “replication, recombination and repair” (2,357, 17.0%), “transcription” (2,268, 16.3%), “function unknown” (1,647, 11.8%), “signal transduction mechanisms” (1,610, 11.6%), “carbohydrate transport and metabolism” (1553, 11.2%), “post-translational modification, protein turnover, chaperon” (1,474, 10.6%), and “inorganic ion transport and metabolism” (1,360, 9.78%), whereas “nuclear structure” (12, 0.09%) was the smallest group (Fig. S6).

KEGG pathway analysis revealed that diverse pathways were represented in the transcriptome dataset, with 25,938 unigenes assigned to 259 specific pathways. Among these pathways, “signal transduction”, “infectious diseases”, “immune system”, “global map for metabolism”, and “cell communication” were the five most-represented pathways (Fig. S7). Some important pathways involved in gonad development were also identified, including “endocrine system”, “nervous system”, and “endocrine and metabolic diseases”.

### Gonad-development-responsive metabolites and unigenes in *C. nasus*

**Figure 2:**
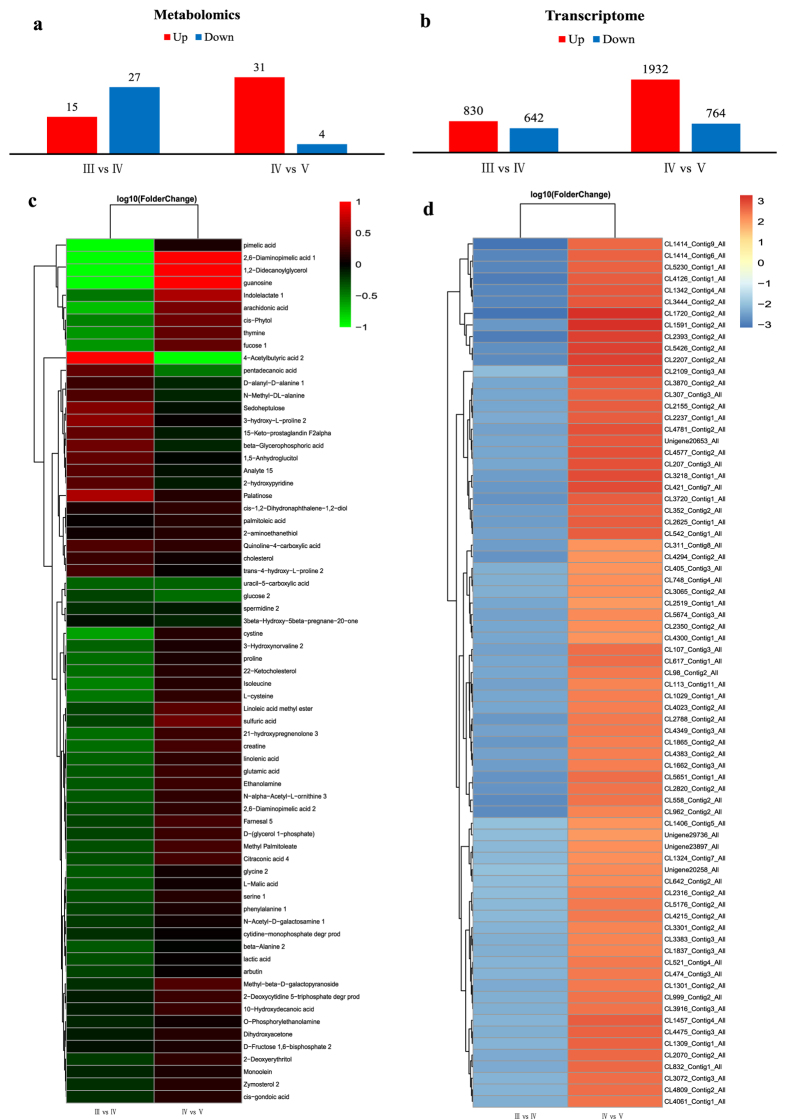
Screening of differentially expressed metabolites and unigenes during ovarian development. (**a**) Differentially expressed metabolites during ovarian development. (**b**) Differentially expressed unigenes during ovarian development. (**c**) Heat map of differentially expressed metabolites. (**d**) Heat map of differentially expressed unigenes (fold-change ≥6).

Fifteen metabolites were significantly up-regulated and 27 were significantly down-regulated from stage III to stage IV, and 31 metabolites were significantly up-regulated and four were down-regulated from stage IV to stage V (Fig. 2a). The up-regulated and down-regulated metabolites are listed in supplementary Table S4. The metabolites included metabolic enzymes, and steroid-synthesis-related metabolites, such as 21-hydroxypregnenolone, lecithin, and cholesterol.

Unigene expression was calculated by the RPKM method, and differentially expressed genes were identified with reference to Audic^20^. A total of 830 genes were significantly up-regulated and 642 were significantly down-regulated from stage III to stage IV, and 1,932 genes were significantly up-regulated and 764 were down-regulated from stage IV to stage V (Fig. 2b). The up-regulated and down-regulated genes are listed in supplementary Table D2. These genes included metabolic genes, enzymes, and other steroid-related genes, such as GTPase, threonine-protein kinase, and aromatase-family genes. These genes showed different expression patterns during ovarian development, implying that they may play important roles in the associated physiological processes.

### Key pathways related to asynchronous ovarian development

**Figure 3:**
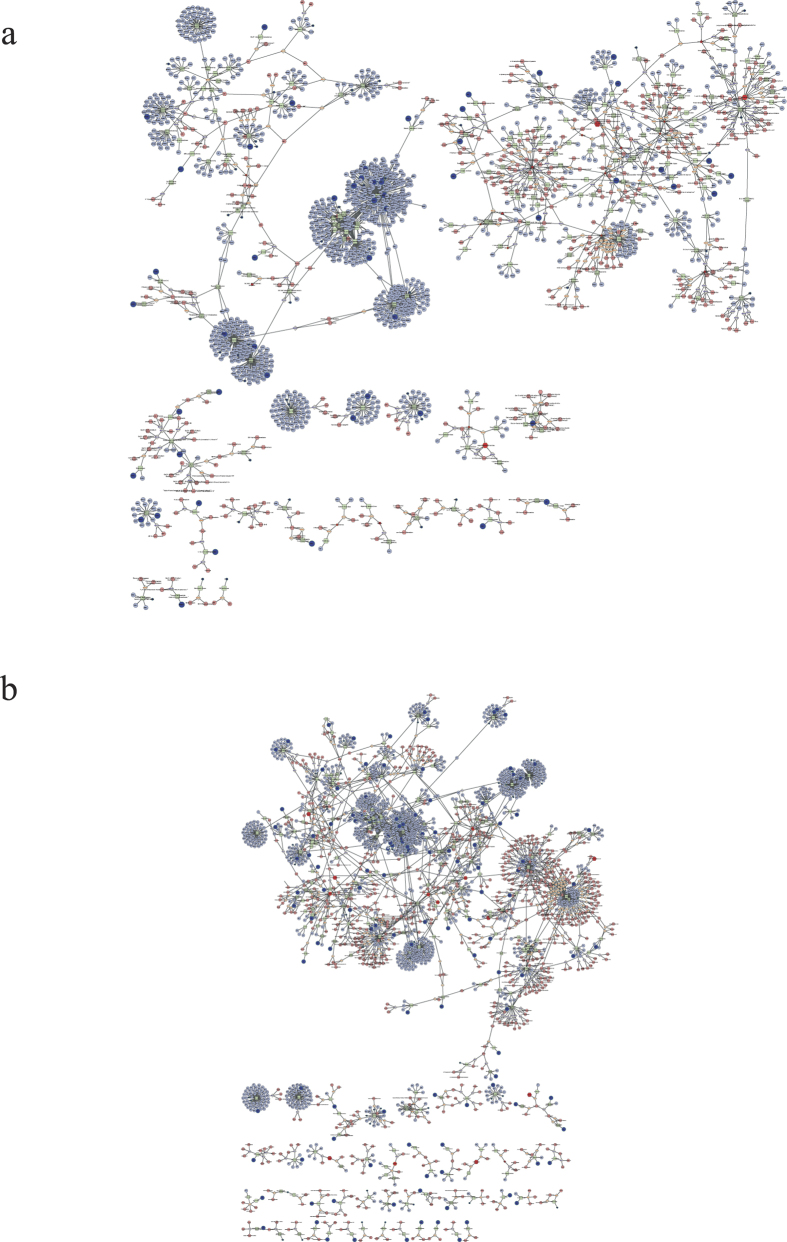
Visualization of enriched metabolome and gene expression profiles using MetScape 3. (**a**) Enriched pathways from stages III to IV, and (**b**) from stages IV to V.

**Figure 4:**
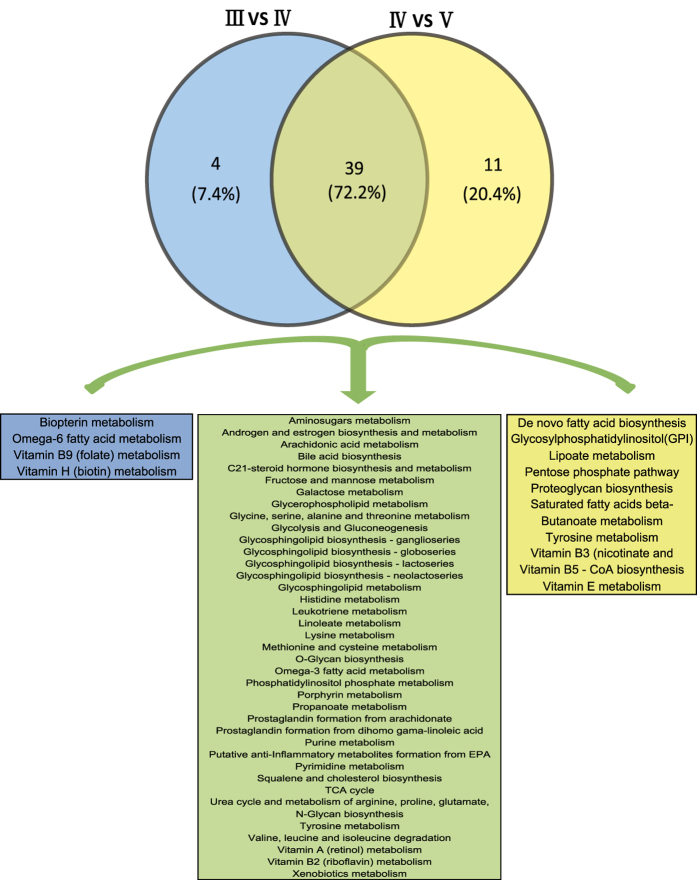


MetScape 3 was used to visualize and interpret the metabolomic and gene expression profiling data^21^. It allowed us to build and analyze networks of genes and compounds, identify enriched pathways from the differential expression profiling data, and visualize changes in metabolite data. The pathways enriched from stage III to stage IV, and from stage IV to stage V are shown in Fig. 3a,b, respectively. Forty-three signaling pathways had important functions from stage III to stage IV of ovarian development, containing 71 key genes and 41 metabolites. Fifty signaling pathways played important roles from stage IV to stage V, including 189 key genes and 22 metabolites. Among the above signaling pathways, 39 were involved in ovarian development from stage III to stage V (Fig. 4). Estrogen and steroid-hormone-biosynthesis pathways including “steroid hormone biosynthesis and metabolism”, “prostaglandin formation”, and “androgen and estrogen biosynthesis and metabolism” are known to play important roles in ovarian development. Other pathways were involved in carbohydrate, lipid, and amino acid metabolism, including “glycolysis and gluconeogenesis”, “histidine metabolism”, “lysine metabolism”, and “omega-3 fatty acid metabolism”.

## Discussion

Ovarian development in fish is a complex biological process affected by genetic and environmental factors^22^. Environmental factors include light, temperature, nutrients, salinity, water flow, and water quality^23^,^24^,^25^, while genetic factors are mainly associated with the different genetic backgrounds of individuals^26^,^27^. However, whether genetic or environmental factors are predominantly responsible for population-asynchronous ovarian development remains unclear. We investigated this issue in 200 wild *C. nasus* collected from the Yangtze River. The ratio of stage III:stage IV:stage V:stage VI in these wild fish was 4:3:2:1 (Fig. S8). We compared this with 210 *C. nasus* collected from artificial breeding ponds with consistent environmental conditions such as light, temperature, water quality, and food. The ratio of stage III:stage IV:stage V among these cultured fish was 3:3:4 (Fig. S8). The stage III:stage IV:stage V ratios were similar in wild and artificially cultured *C. nasus*, but stage VI fish were only found in the wild *C. nasus* population. This result suggested that population-asynchronous ovary development occurs under both natural and artificial conditions, even though the environmental conditions were consistent, implying that genetic factors are important for determining population-asynchronous ovary development. In this study, we further explored the genetic factors, including genes and metabolites, affecting asynchronous ovary development using fish cultured under consistent artificial conditions, to eliminate any potential environmental impacts.

Forty-three signaling pathways containing 71 key genes and 41 metabolites appeared to have key functions during ovary development from stages III to IV, while 50 signaling pathways containing 189 key genes and 22 metabolites were involved from stage IV to V (Fig. 4). Thirty-nine of the pathways were involved from stage III to stage V, including pathways related to the metabolism of hormones, amino acids, carbohydrates, and lipids (Fig. 4). Among these pathways, steroid metabolism has been reported to play an important role in ovarian development. Steroids are lipophilic, low-molecular-weight compounds derived from cholesterol that play a number of important physiological roles. Steroid hormones are mainly synthesized by endocrine glands such as the gonads (testis and ovary), adrenals, and (during gestation) by the fetoplacental unit, and are then released into the blood circulation. In our study, three key pathways reflected the changes in metabolite levels consistent with changes in transcript abundance as the ovary developed from stage III to stage IV: “squalene and cholesterol biosynthesis”, “steroid hormone biosynthesis”, and “arachidonate metabolism and prostaglandin formation” (Fig. 5).

Squalene and cholesterol biosynthesis: from acetate to cholesterol. Cholesterol can be synthesized from acetate in all steroid-producing tissues. The 27-carbon skeleton of cholesterol is derived from acetyl-CoA through a series of reactions. (1) Mevalonate is produced by condensation of three molecules of acetyl-CoA via mevalonate kinase (MVK), which is a rate-limiting enzyme in the mevalonate pathway. MVK transfers the phosphate group at the ATP-γ-position to the 5-hydroxy position of mevalonate to form mevalonate-5-phosphate and release ADP. In our studies, MVK expression was significantly increased during ovarian development. (2) Squalene is a 30-carbon linear structure that undergoes cyclization to yield (3) lanosterol via lanosterol synthase (LS), which is a key enzyme in cholesterol (animals) and terpenoid (fungi) syntheses. LS catalyzes(S)-squalene 2,3-epoxide cyclization to lanosterol and generates cholesterol in animals. LS levels and activity thus affect cholesterol production. In this study, LS gene expression was significantly up-regulated during ovarian development. (4) Cholesterol is produced after the removal of three carbons. This important metabolite was significantly increased from stages III to IV, but not from stages IV to V. Accordingly, previous reports have indicated that cholesterol accumulates from stages III to IV, while steroids accumulate from stages IV to V^28^,^29^. In summary, expression of the cholesterol synthesis pathway is increased during ovarian development.

Steroid hormone biosynthesis: from cholesterol to progestins, androgens, and estrogens. All steroid hormones are synthesized from cholesterol. Cholesterol is converted to pregnenolone via a rate-limiting step subject to hormonal control by adrenocorticotropic hormone in the adrenals and luteinizing hormone (LH) in the gonads, catalyzed by the cholesterol side-chain cleavage enzyme P-450 scc (20,22-desmolase; 20,22-lyase). Pregnenolone is further metabolized to progesterone by 3-β-hydroxysteroid dehydrogenase and δ-5 oxygen steroid isomerase, and to 17α-hydroxylase pregnenolone by the addition of 17α-hydroxy by 17α- hydroxylase. Our results suggested that *SULT2B1* and *CYP17A* (steroid 17α-hydroxylase/17, 20-lyase) expression were significantly increased during ovary development. As reported previously^30^,^31^, the sulfotransferase family cytosolic 2B member 1 isoform (encoded by *SULT2B1*) sulfonates cholesterol in humans and mice, while the B1a isoform sulfonates pregnenolone^30^,^32^,^33^. Cytochrome P450 s play critical roles in the metabolism of various bioactive compounds, including the biosynthesis of steroid hormones in chordata. CYP17 is localized in endoplasmic reticulum membranes of steroidogenic cells^34^ and catalyzes the 1-α-hydroxylation of δ4-C21 steroids (progesterone derivatives) and δ5-C21 steroids (pregnenolone derivatives), as well as C19-steroids, a key branch point in steroid hormone biosynthesis, via the 17, 20-lyase reaction^35^. Depending on CYP17 activity, the steroid hormone biosynthesis pathway is directed to the formation of either mineralocorticoids and glucocorticoids or sex hormones. In the present study, CYP17A expression was significantly increased^36^, indicating increased estrogen synthesis and reduced cortisol synthesis during the ovarian development process.

Arachidonate metabolism and prostaglandin formation: from arachidonate to prostaglandin. Prostaglandins act on neuroendocrine cells to increase the release of LH-releasing hormone, stimulating pituitary secretion of LH and follicle-stimulating hormone, which in turn stimulate ovulation^37^,^38^,^39^. The biosynthesis of prostaglandin E2 (PGE2) from arachidonic acid is catalyzed in a sequential reaction by PGH synthase forming the endoperoxide PGG2, followed by PGH2 by reduction. PGE synthase (EC5.3.99.3) subsequently converts PGH2 into PGE2^40^,^41^. PGE synthase gene expression was significantly increased at stage V in the current study.

Arachidonate can be synthesized from lecithin and a C20:0 fatty acid (e.g., linoleic acid). In this study, the lecithin content was significantly increased from stages IV to V, but not from stages III to IV. This increase at stage V may be related to the fact that prostaglandins stimulate ovulation.

15-Lipoxygenases (LOXs) are lipid-peroxidizing enzymes that catalyze the stereoselective introduction of molecular dioxygen at carbon 15 (C-15) of arachidonic acid^42^. With linoleic acid as a substrate, oxygen is introduced at C-13. The primary products of the 15-LOX reaction are n-6 hydroperoxy fatty acids containing a Z, E-conjugated diene system^43^. In cellular systems, the fatty acid hydroperoxides are rapidly reduced to the corresponding hydroxy derivatives^44^,^45^. Glutathione peroxidase (GPX) is a key enzyme used to remove reactive oxygen species^46^,^47^ and thus protect cells from oxidative damage by deoxygenizing hydrogen peroxide, organic hydroperoxides, and lipid peroxides^48^. GPX3 knockout significantly reduced the antioxidant capacity, while its over expression significantly enhanced it. In this study, arachidonate 15-lipoxygenase 15B expression decreased, while GPX3 expression slightly increased in stage V ovaries, indicating that oxidation of the arachidonic acid metabolic pathway was inhibited during ovarian development.

In conclusion, we used a combination of transcriptomic and metabolomic methods to identify the key signaling pathways and gene expression profiles associated with asynchronous ovarian development. We found 43 signaling pathways that played important functions from stages III to IV, and 50 that were involved from stages IV to V of ovarian development, including 39 signaling pathways involved from stages III to V. These results suggest that the “squalene and cholesterol biosynthesis”, “steroid hormone biosynthesis”, and “arachidonate metabolism and prostaglandin formation” pathways are key pathways regulating asynchronous ovarian development in *C. nasus* and other migratory fish species.

## Data Availability. 

Raw sequencing data is available through the NCBI Sequence Read Archive under Project Accession PRJNA317021 (http://www.ncbi.nlm.nih.gov/).

## Additional Information

**How to cite this article**: Xu, G. *et al*. Integrated application of transcriptomics and metabolomics yields insights into population-asynchronous ovary development in *Coilia nasus. Sci. Rep.* **6**, 31835; doi: 10.1038/srep31835 (2016).

## Supplementary Material

Supplementary Information

Supplementary Dataset 1

Supplementary Dataset 2

## Figures and Tables

**Figure 5:**