# Supplementary material for: Integrated application of transcriptomics and metabolomics yields insights into population-asynchronous ovary development in Coilia nasus
Source: Sci Rep. 2016 Aug 22;6:31835. doi: 10.1038/srep31835 (PMC4992829; doi:10.1038/srep31835)
Supplement: Supplementary Information [file srep31835-s1.pdf]

Integrated application of transcriptomics and metabolomics yields insights into population-asynchronous ovary development in *Coilia nasus*

GangchunXu[a,b], Fukuan Du[b], Yan Li[b], ZhijuanNie[b] & PaoXu[a,b,*]

[a]Wuxi Fisheries College, Nanjing Agricultural University, Wuxi, Jiangsu, 214081, China

[b]Key Laboratory of Freshwater Fisheries and Germplasm Resources Utilization, Ministry of Agriculture, Freshwater Fisheries Research Center, Chinese Academy of Fishery Sciences, Wuxi, Jiangsu, 214081, China

*Corresponding author. Tel.: +86-0510-85557959; fax: +86-0510-85390029

Email: xup13806190669@163.com

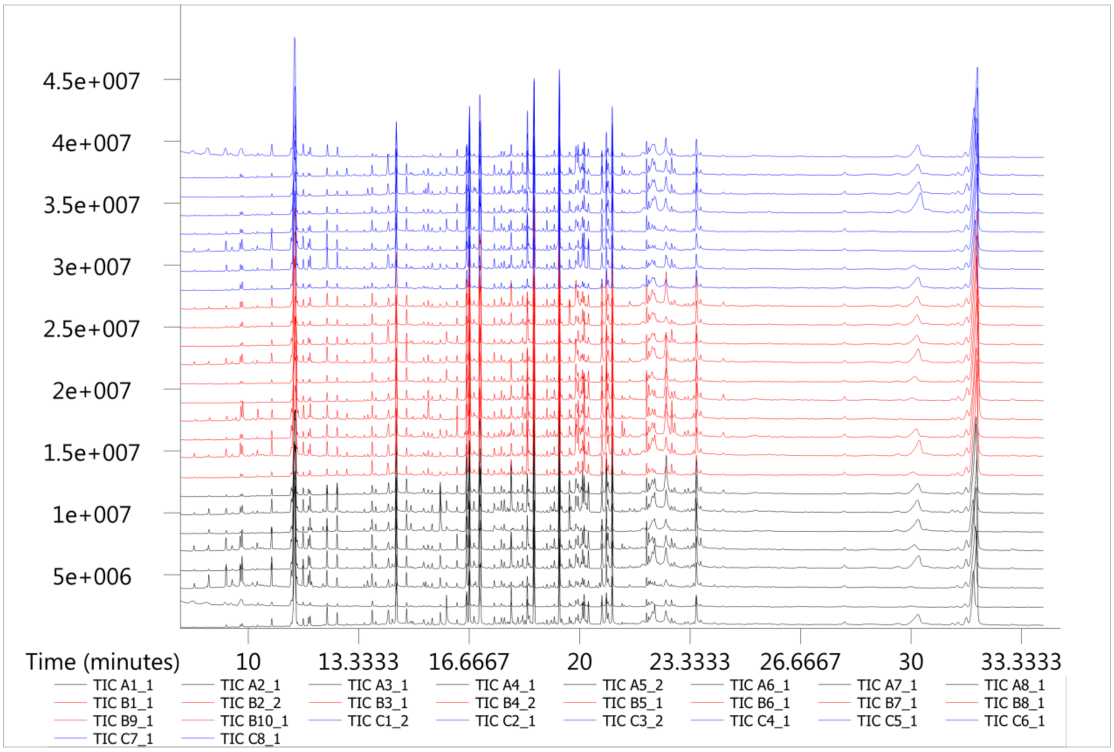
4.5e+007
4e+007
3.5e+007
3e+007
2.5e+007
2e+007
1.5e+007
1e+007
5e+006
Time (minutes)
10
13.3333
16.6667
20
23.3333
26.6667
30
33.3333
TIC A1_1
TIC A2_1
TIC A3_1
TIC A4_1
TIC A5_2
TIC A6_1
TIC A7_1
TIC A8_1
TIC B1_1
TIC B2_2
TIC B3_1
TIC B4_2
TIC B5_1
TIC B6_1
TIC B7_1
TIC B8_1
TIC B9_1
TIC B10_1
TIC C1_2
TIC C2_1
TIC C3_2
TIC C4_1
TIC C5_1
TIC C6_1
TIC C7_1
TIC C8_1

Fig. S1

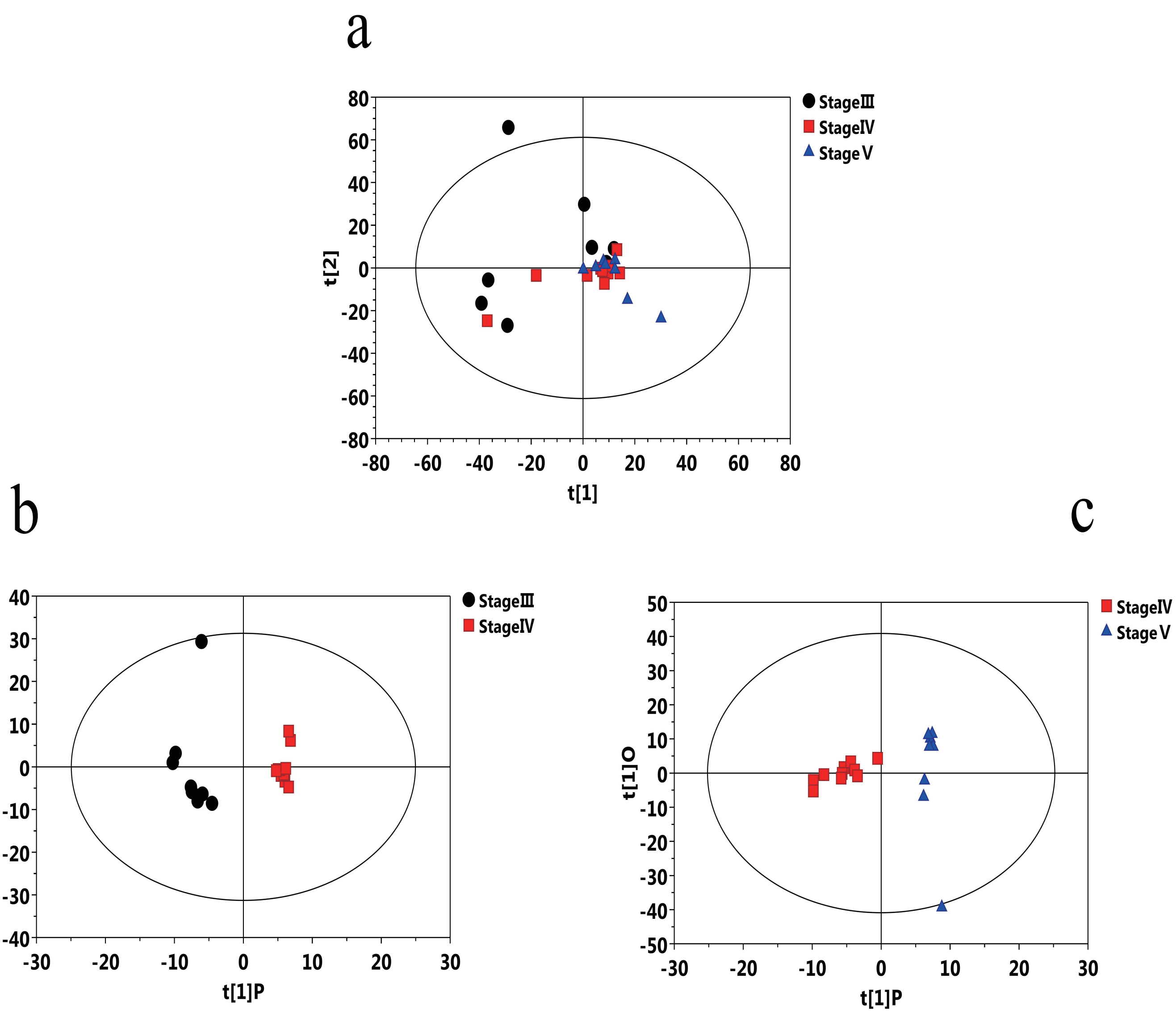
a
StageⅢ
StageⅣ
Stage V
t[2]
t[1]
b
StageⅢ
StageⅣ
t[1]O
t[1]P
c
StageⅣ
Stage V
t[1]O
t[1]P

Fig. S2

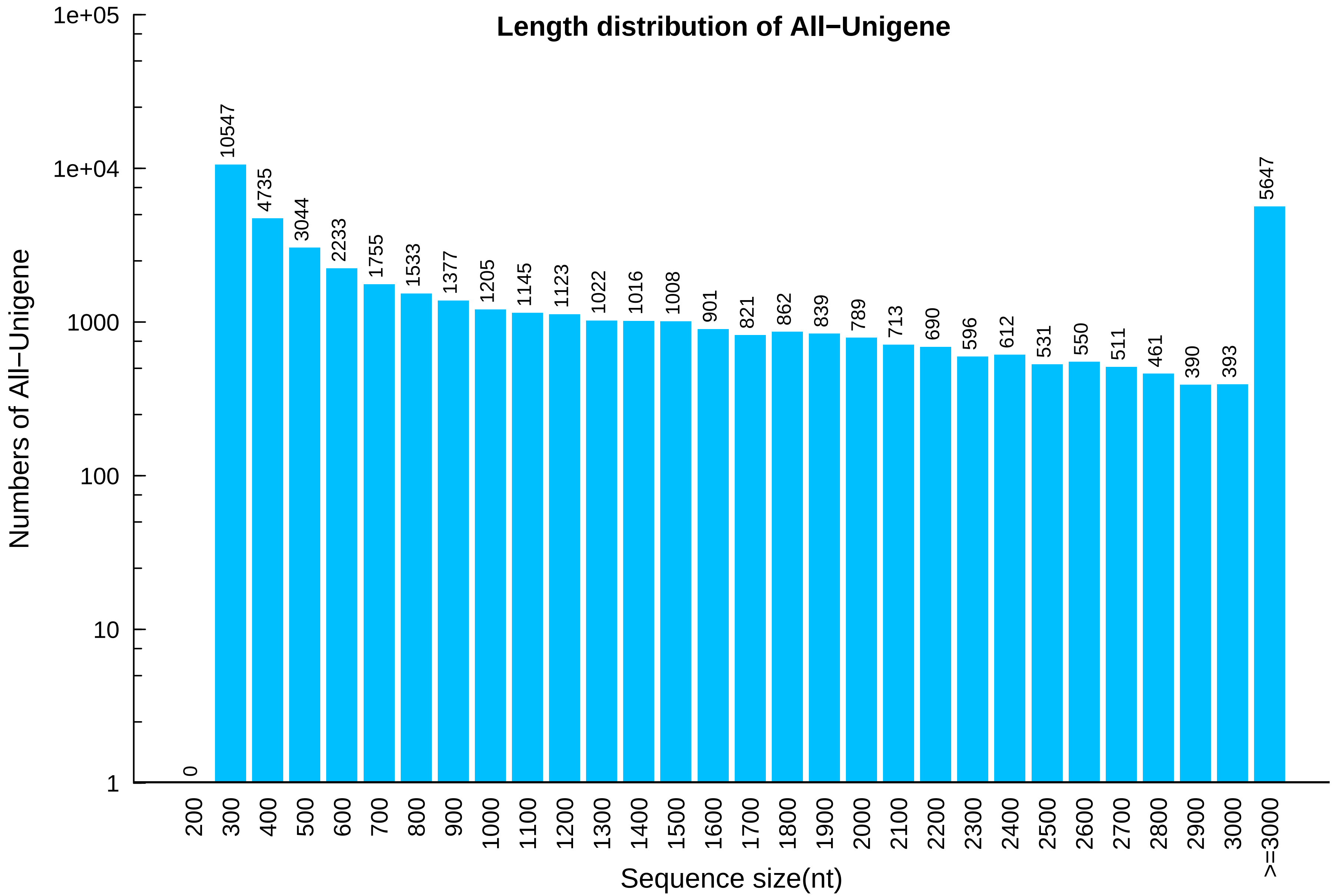

Length distribution of All−Unigene
Numbers of All−Unigene
Sequence size(nt)
1e+05
1e+04
1000
100
10
1
0
10547
4735
3044
2233
1755
1533
1377
1205
1145
1123
1022
1016
1008
901
821
862
839
789
713
690
596
612
531
550
511
461
390
393
5647
200
300
400
500
600
700
800
900
1000
1100
1200
1300
1400
1500
1600
1700
1800
1900
2000
2100
2200
2300
2400
2500
2600
2700
2800
2900
3000
>=3000

Fig. S3

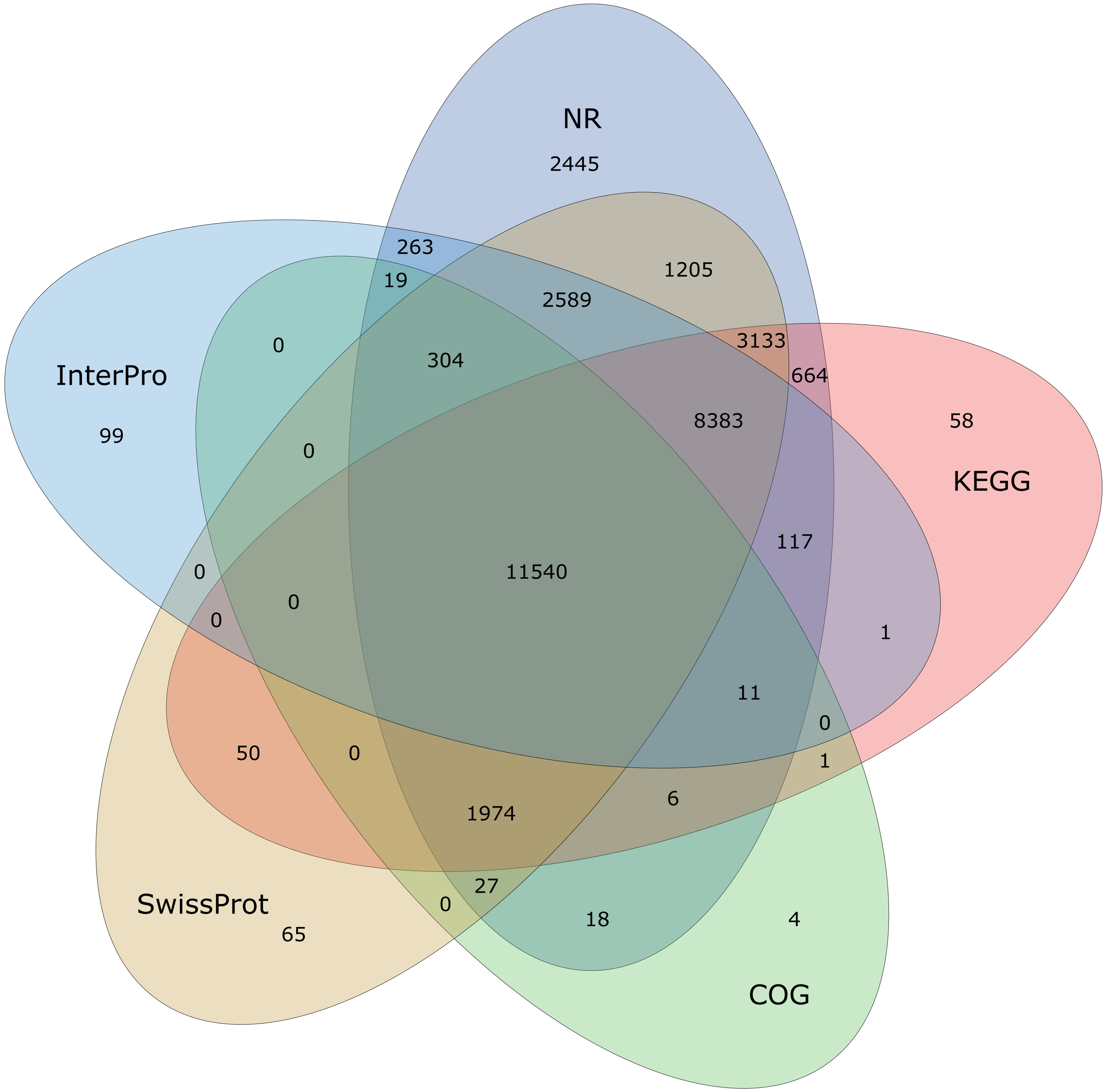
NR
2445
263
19
1205
2589
3133
664
0
304
InterPro
99
8383
58
0
KEGG
117
0
11540
0
0
1
11
0
50
0
1
6
1974
27
SwissProt
0
18
4
65
COG

Fig. S4

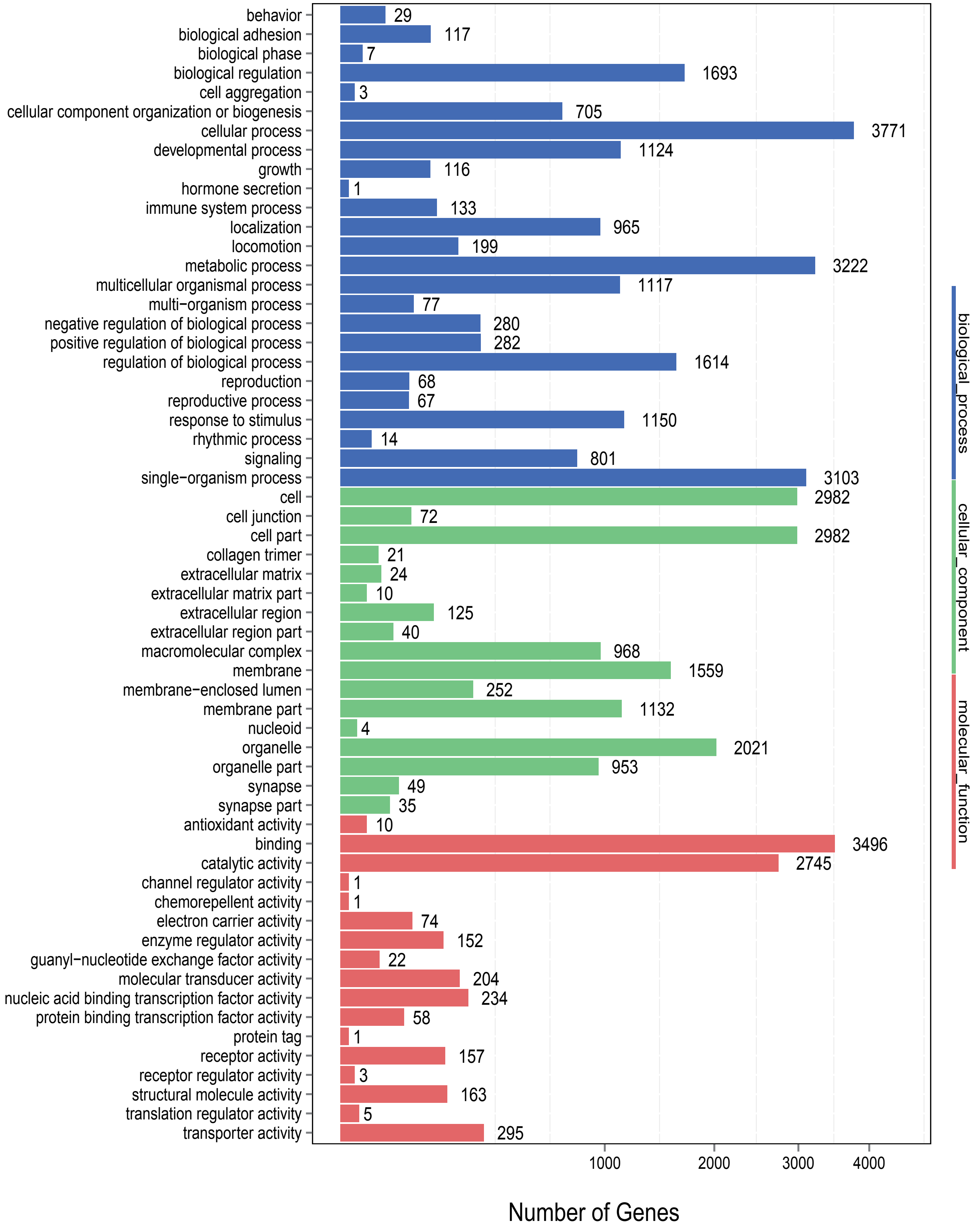
behavior
biological adhesion
biological phase
biological regulation
cell aggregation
cellular component organization or biogenesis
cellular process
developmental process
growth
hormone secretion
immune system process
localization
locomotion
metabolic process
multicellular organismal process
multi−organism process
negative regulation of biological process
positive regulation of biological process
regulation of biological process
reproduction
reproductive process
response to stimulus
rhythmic process
signaling
single−organism process
cell
cell junction
cell part
collagen trimer
extracellular matrix
extracellular matrix part
extracellular region
extracellular region part
macromolecular complex
membrane
membrane−enclosed lumen
membrane part
nucleoid
organelle
organelle part
synapse
synapse part
antioxidant activity
binding
catalytic activity
channel regulator activity
chemorepellent activity
electron carrier activity
enzyme regulator activity
guanyl−nucleotide exchange factor activity
molecular transducer activity
nucleic acid binding transcription factor activity
protein binding transcription factor activity
protein tag
receptor activity
receptor regulator activity
structural molecule activity
translation regulator activity
transporter activity
29
117
7
1693
3
705
3771
1124
116
1
133
965
199
3222
1117
77
280
282
1614
68
67
1150
14
801
3103
2982
72
2982
21
24
10
125
40
968
1559
252
1132
4
2021
953
49
35
10
3496
2745
1
1
74
152
22
204
234
58
1
157
3
163
5
295
1000
2000
3000
4000
Number of Genes
biological_process
cellular_component
molecular_function

Fig. S5

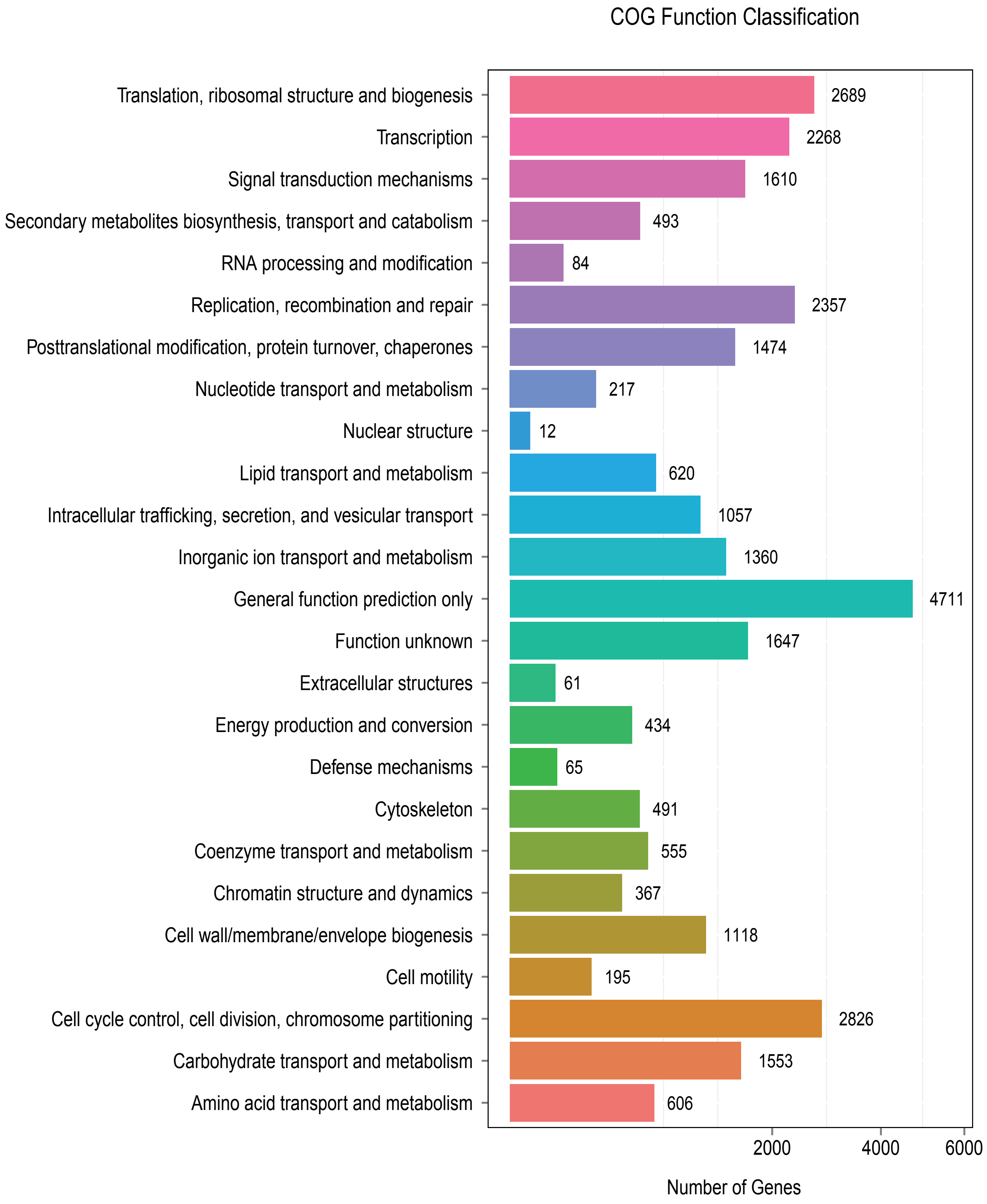
COG Function Classification
Translation, ribosomal structure and biogenesis
Transcription
Signal transduction mechanisms
Secondary metabolites biosynthesis, transport and catabolism
RNA processing and modification
Replication, recombination and repair
Posttranslational modification, protein turnover, chaperones
Nucleotide transport and metabolism
Nuclear structure
Lipid transport and metabolism
Intracellular trafficking, secretion, and vesicular transport
Inorganic ion transport and metabolism
General function prediction only
Function unknown
Extracellular structures
Energy production and conversion
Defense mechanisms
Cytoskeleton
Coenzyme transport and metabolism
Chromatin structure and dynamics
Cell wall/membrane/envelope biogenesis
Cell motility
Cell cycle control, cell division, chromosome partitioning
Carbohydrate transport and metabolism
Amino acid transport and metabolism
2689
2268
1610
493
84
2357
1474
217
12
620
1057
1360
4711
1647
61
434
65
491
555
367
1118
195
2826
1553
606
2000
4000
6000
Number of Genes

Fig. S6

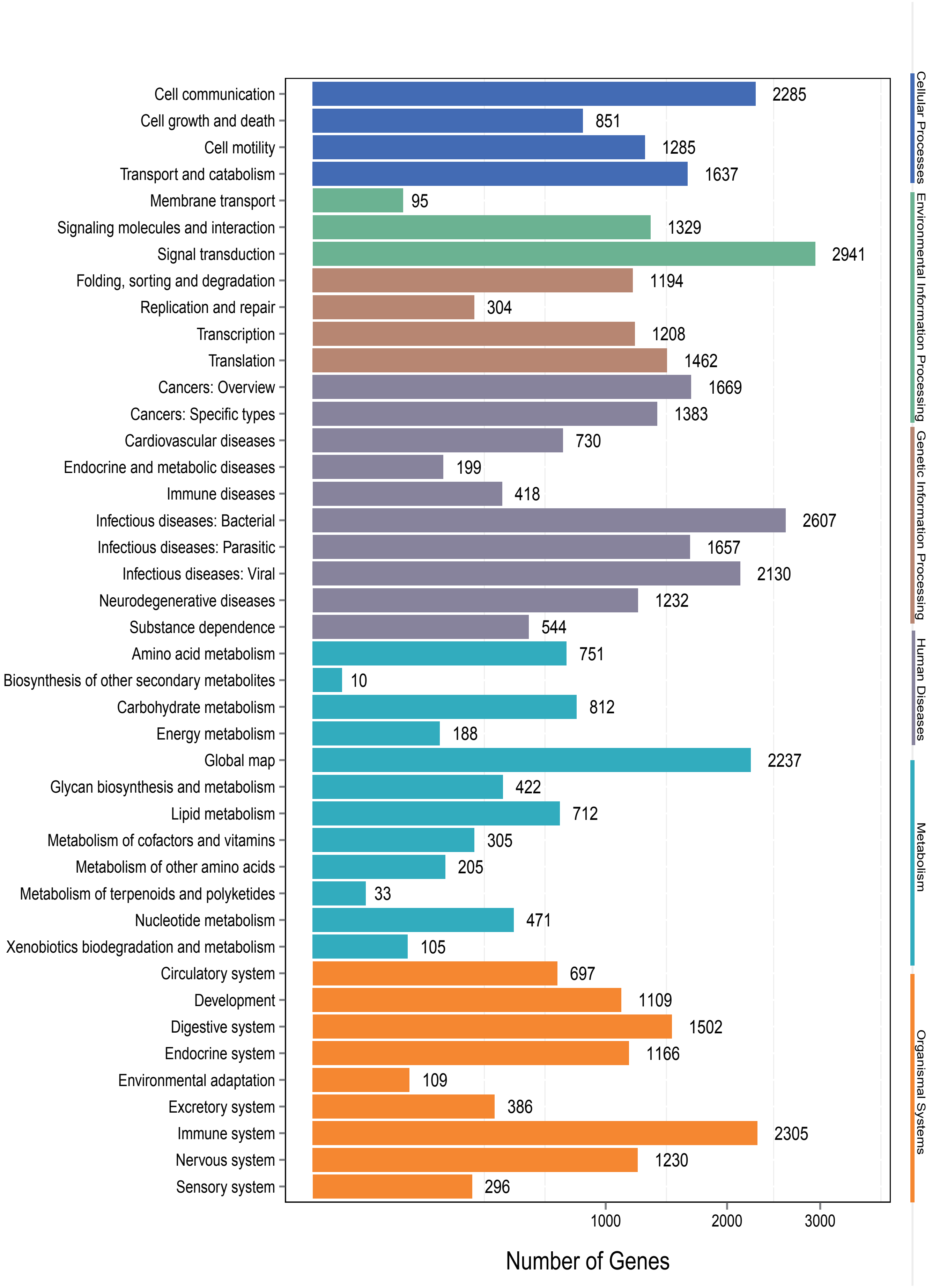
Cell communication
Cell growth and death
Cell motility
Transport and catabolism
Membrane transport
Signaling molecules and interaction
Signal transduction
Folding, sorting and degradation
Replication and repair
Transcription
Translation
Cancers: Overview
Cancers: Specific types
Cardiovascular diseases
Endocrine and metabolic diseases
Immune diseases
Infectious diseases: Bacterial
Infectious diseases: Parasitic
Infectious diseases: Viral
Neurodegenerative diseases
Substance dependence
Amino acid metabolism
Biosynthesis of other secondary metabolites
Carbohydrate metabolism
Energy metabolism
Global map
Glycan biosynthesis and metabolism
Lipid metabolism
Metabolism of cofactors and vitamins
Metabolism of other amino acids
Metabolism of terpenoids and polyketides
Nucleotide metabolism
Xenobiotics biodegradation and metabolism
Circulatory system
Development
Digestive system
Endocrine system
Environmental adaptation
Excretory system
Immune system
Nervous system
Sensory system
2285
851
1285
1637
95
1329
2941
1194
304
1208
1462
1669
1383
730
199
418
2607
1657
2130
1232
544
751
10
812
188
2237
422
712
305
205
33
471
105
697
1109
1502
1166
109
386
2305
1230
296
1000
2000
3000
Number of Genes
Cellular Processes
Environmental Information Processing
Genetic Information Processing
Human Diseases
Metabolism
Organismal Systems

Fig. S7

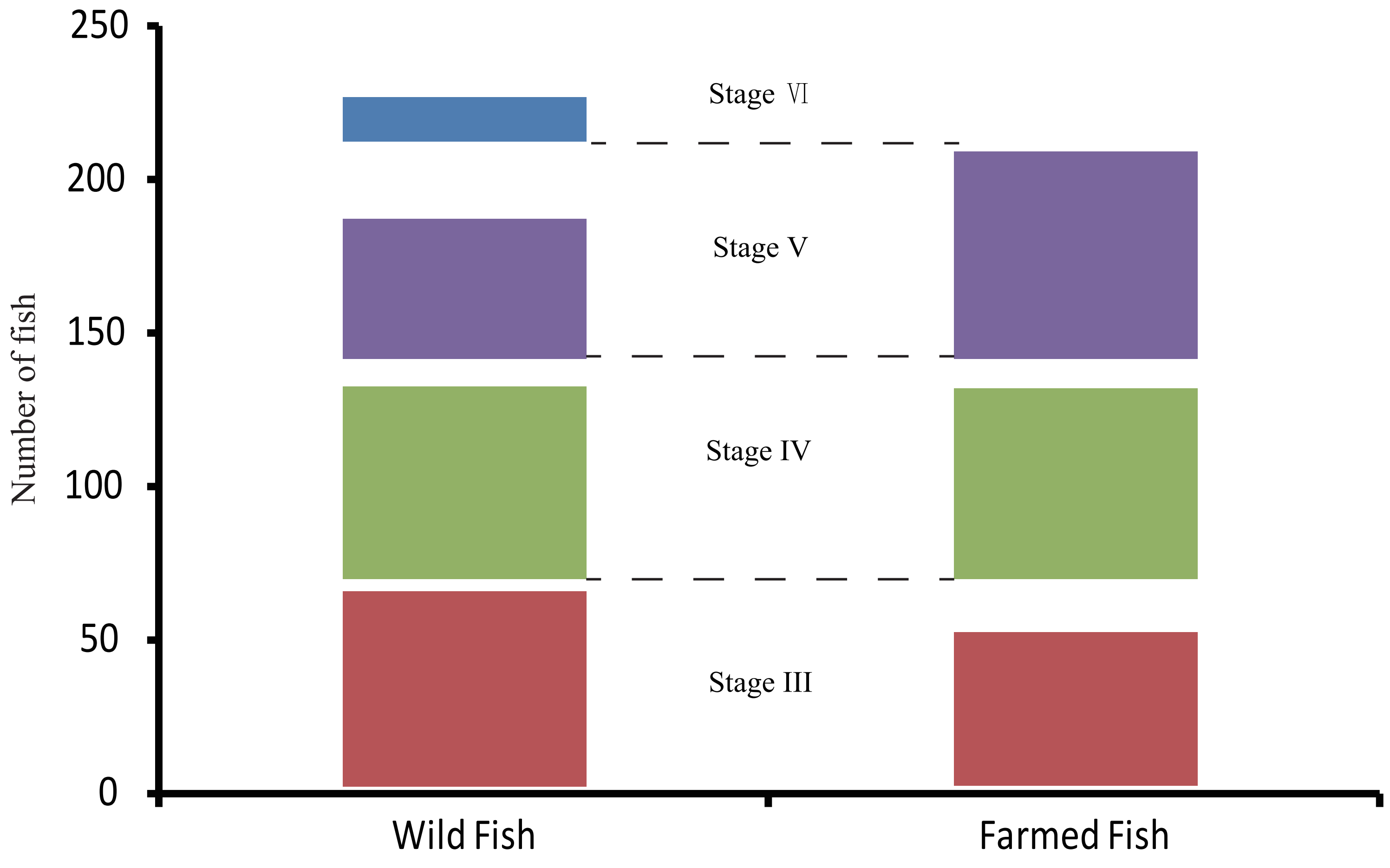

250
200
150
100
50
0
Number of fish
Stage Ⅵ
Stage V
Stage IV
Stage III
Wild Fish
Farmed Fish

Fig. S8

Table S1. Sequencing reads after filtering.

Sample	Total Raw Reads(Mb)	Total Clean Reads(Mb)	Total Clean Bases(Gb)	Clean Reads Q20(%)	Clean Reads Q30(%)	Clean Reads Ratio(%)
Stag Ⅲ	65.87	65.75	6.57	97.62	94	99.82
Stage Ⅳ	65.86	65.75	6.57	97.27	93.41	99.84
Stage Ⅴ	65.86	65.70	6.57	97.72	94.34	99.75

Table S2. Quality metrics of Unigenes.

Sample	Total Number	Total Length	Mean Length	N50	N70	N90	GC(%)
Stage_III	39044	46883889	1200	2157	1351	476	50.94
Stage_IV	40035	48017894	1199	2172	1360	469	50.93
Stage_V	45058	51009318	1132	2062	1261	433	50.55
All-Unigene	47049	63913175	1358	2472	1587	568	50.78

Table S3. Annotation result of each functional database.

Values	Total	Nr-Annotated	Nt-Annotated	Swissprot-Annotated	KEGG-Annotated	COG-Annotated	Interpro-Annotated	GO-Annotated	Overall
Number	47,049	32,698	33,287	29,270	25,938	13,904	23,326	6,735	37,853
Percentage	100%	69.50%	70.75%	62.21%	55.13%	29.55%	49.58%	14.31%	80.45%

Table S4. Differentially expressed metabolites from stages III to IV and stages IV to V.

Stage Ⅲ vs Stage Ⅳ

Peak	R.T.	Mass	P-Value	Q-Value	LOG_Fold Change
proline	11.815,0	142	0.009063442	0.217888144	-1.402825441
glycine 2	11.8654,0	174	0.085581427	0.361153077	-1.181902902
serine 1	12.3761,0	204	0.008470857	0.216503895	-0.972226736
L-Malic acid	13.8473,0	147	0.021551625	0.275000094	-1.125527525
Isoleucine	11.6577,0	158	0.071524817	0.353820739	-1.626863442
2-hydroxypyridine	8.7425,0	152	0.098559128	0.366238181	1.165742392
phenylalanine 1	15.5473,0	218	0.00623234	0.212273518	-0.890266027
N-Acetyl-D-galactosamine 1	19.6032,0	202	0.078886679	0.357949847	-0.709333141
spermidine 2	20.6769,0	174	0.070684742	0.353301714	-0.643114148
glutamic acid	15.2952,0	246	0.028594259	0.298436123	-1.192415285
trans-4-hydroxy-L-proline 2	14.3258,0	230	0.035711406	0.314797759	0.87692299
fucose 1	16.3694,0	117	0.004349258	0.20896919	-1.8651319
guanosine	25.2928,0	324	0.03648781	0.316280141	-23.05076705
3-Hydroxynorvaline 2	12.6826,0	219	0.005610714	0.211418058	-1.297983975
1,5-Anhydroglucitol	17.6619,0	217	0.006857111	0.212982231	1.253037672
Palatinose	25.9132,0	204	0.001864201	0.19760262	2.135899848
2,6-Diaminopimelic acid 2	17.7219,0	174	0.081064229	0.35904337	-0.999402901
15-Keto-prostaglandin F2alpha	24.0741,0	132	0.083499922	0.360206234	1.32436945

beta-Alanine 2	13.2575,0	218	0.07830879	0.357650597	-1.091939557
arachidonic acid	22.2035,0	91	0.012513349	0.236767946	-2.458039304
thymine	12.9778,0	70	0.081643725	0.359325635	-1.965821221
Sedoheptulose	18.3627,0	147	0.002054907	0.19760262	1.683507677
arbutin	23.3773,0	259	0.072468656	0.354391272	-0.881708611
D-alanyl-D-alanine 1	15.2563,0	188	0.043268104	0.327392256	0.784103074
beta-Glycerophosphoric acid	28.8494,0	129	0.000882004	0.19760262	1.403452819
1,2-Didecanoylglycerol	27.9806,0	229	0.042215009	0.325855341	-24.57885865
2,6-Diaminopimelic acid 1	10.714,0	38	0.05417204	0.340344682	-22.00757698
Linoleic acid methyl ester	23.1808,0	36	0.051035243	0.337092071	-1.377839971
pimelic acid	15.1766,0	129	0.015576268	0.247863106	-24.61722628
22-Ketocholesterol	22.993,0	173	0.068500418	0.351900234	-1.358703267
cystine	15.4379,0	194	0.015094211	0.24635684	-2.015833321
pentadecanoic acid	21.4591,0	117	0.027718504	0.295997208	1.300223132
4-Acetylbutyric acid 2	12.082,0	254	0.069579149	0.352601961	18.4492471
3-hydroxy-L-proline 2	10.8534,0	57	0.012164457	0.235232113	1.729588112
lactic acid	24.1098,0	71	0.007400921	0.213504715	-0.954394268
proline	14.7309,0	196	0.090317647	0.363161217	2.357004895
proline	15.0645,0	142	0.015022788	0.246127065	-1.151350026
linolenic acid	22.8661,0	215	0.08486821	0.360833321	-1.227938803
cytidine-monophosphate degrprod	22.2661,0	217	0.042470415	0.326233745	-0.659975872
cholesterol	31.9751,0	129	0.016501846	0.250553587	0.775723988

N-Methyl-DL-alanine	7.80357,0	83	0.087013091	0.361780755	0.975923895
Analyte 15	7.98229,0	56	0.059826959	0.345471034	1.117657611
**Stage Ⅳ vs Stage Ⅴ**					
palmitoleic acid	19.2347,0	129	0.0442207	0.5941233	0.499952
O-Phosphorylethanolamine	16.9808,0	172	0.0584963	0.5941233	0.2024206
Ethanolamine	11.3484,0	174	0.0469648	0.5941233	0.7896578
Methyl-beta-D -galactopyranoside	17.5568,0	204	0.0312816	0.5941233	0.9715079
L-cysteine	14.6751,0	220	0.0265449	0.5941233	0.5619707
Monoolein	25.1449,0	129	0.0982566	0.5941233	0.5034016
Farnesal 5	18.4489,0	218	0.0944167	0.5941233	0.7995868
fucose 1	16.3694,0	117	0.0895672	0.5941233	1.3156056
glucose 2	17.8178,0	155	0.0292614	0.5941233	-1.4004448
sulfuric acid	10.1327,0	73	0.048656	0.5941233	1.3541212
Citraconic acid 4	12.4274,0	243	0.0385184	0.5941233	0.9115147
Linoleic acid methyl ester	23.0768,0	55	0.0057559	0.5941233	1.0923236
O-Phosphorylethanolamine	18.8337,0	100	0.0430098	0.5941233	0.367046
D-(glycerol 1-phosphate)	16.3064,0	89	0.0268411	0.5941233	0.7687486
2-aminoethanethiol	20.8701,0	357	0.0220732	0.5941233	0.4426002
2-Deoxyerythritol	24.2876,0	205	0.0484263	0.5941233	0.6147191
uracil-5-carboxylic acid	16.8518,0	287	0.0649422	0.5941233	-1.2474519
N-alpha-Acetyl-L-ornithine 3	16.2497,0	57	0.0589817	0.5941233	0.6219342
10-Hydroxydecanoic acid	17.324,0	89	0.013891	0.5941233	0.758919

Methyl Palmitoleate	31.2311,0	57	0.0031429	0.5941233	0.8342169
cis-gondoic acid	20.035,0	145	0.0425144	0.5941233	0.4771281
3beta-Hydroxy-5beta-pregnane-20-one	19.6238,0	107	0.0836772	0.5941233	-0.4530409
21-hydroxypregnenolone 3	22.6098,0	157	0.0665026	0.5941233	0.7881963
cis-Phytol	22.5731,0	154	0.0101864	0.5941233	1.4496718
Quinoline-4-carboxylic acid	17.0677,0	82	0.0582681	0.5941233	0.5722321
4-Acetylbutyric acid 2	12.082,0	254	0.0695788	0.5941233	-19.579577
cis-1,2-Dihydronaphthalene-1,2-diol	13.0658,0	191	0.087494	0.5941233	0.6544903
2'-Deoxycytidine 5'-triphosphate degr prod	14.5725,0	199	0.0921169	0.5941233	0.7482912
creatine	22.6587,0	224	0.0662652	0.5941233	0.8645318
D-Fructose 1,6-bisphosphate 2	22.1938,0	318	0.0699251	0.5941233	0.310676
Monoolein	21.8678,0	129	0.0711396	0.5941233	0.3880898
Dihydroxyacetone	18.7595,0	133	0.0997089	0.5941233	0.4022401
Zymosterol	21.1731,0	97	0.0206854	0.5941233	3.9911234
linolenic acid	22.8661,0	215	0.0649968	0.5941233	0.6591066
Indolelactate 1	8.04553,0	130	0.0771691	0.5941233	2.1836071

Figure legends

Figure S1. Total chromatograms of homogenized ovary samples analyzed by GC–MS.

Figure S2. PCA and OPLS-DA analyses of GC–MS date.

Figure S3. Length distribution of unigenes.

Figure S4. Blast analysis of non-redundant unigenes against public databases.

Figure S5. GO categorization of non-redundant unigenes.

Figure S6. COG annotation of putative proteins.

Figure S7. KEGGpathway analysis of unigene function.

Figure S8. Rations of overy development stages in wild and artificial cultured *C. nasus.*
